# Analysis of neuronal injury transcriptional response identifies CTCF and YY1 as co-operating factors regulating axon regeneration

**DOI:** 10.3389/fnmol.2022.967472

**Published:** 2022-08-23

**Authors:** Oshri Avraham, Jimmy Le, Kathleen Leahy, Tiandao Li, Guoyan Zhao, Valeria Cavalli

**Affiliations:** ^1^Department of Neuroscience, Washington University School of Medicine, St. Louis, MO, United States; ^2^Department of Developmental Biology, Washington University School of Medicine, St. Louis, MO, United States; ^3^Center of Regenerative Medicine, Washington University School of Medicine, St. Louis, MO, United States; ^4^Department of Pathology and Immunology, Washington University School of Medicine, St. Louis, MO, United States; ^5^Hope Center for Neurological Disorders, Washington University School of Medicine, St. Louis, MO, United States

**Keywords:** axon regeneration, sensory neurons, transcription factors, bioinformatics analyses, CTCF, YY1, E2F2, dorsal root ganglia

## Abstract

Injured sensory neurons activate a transcriptional program necessary for robust axon regeneration and eventual target reinnervation. Understanding the transcriptional regulators that govern this axon regenerative response may guide therapeutic strategies to promote axon regeneration in the injured nervous system. Here, we used cultured dorsal root ganglia neurons to identify pro-regenerative transcription factors. Using RNA sequencing, we first characterized this neuronal culture and determined that embryonic day 13.5 DRG (eDRG) neurons cultured for 7 days are similar to e15.5 DRG neurons *in vivo* and that all neuronal subtypes are represented. This eDRG neuronal culture does not contain other non-neuronal cell types. Next, we performed RNA sequencing at different time points after *in vitro* axotomy. Analysis of differentially expressed genes revealed upregulation of known regeneration associated transcription factors, including *Jun*, *Atf3* and *Rest*, paralleling the axon injury response *in vivo*. Analysis of transcription factor binding sites in differentially expressed genes revealed other known transcription factors promoting axon regeneration, such as *Myc, Hif1α, Pparγ, Ascl1a, Srf*, and *Ctcf*, as well as other transcription factors not yet characterized in axon regeneration. We next tested if overexpression of novel candidate transcription factors alone or in combination promotes axon regeneration *in vitro*. Our results demonstrate that expression of *Ctcf* with *Yy1* or *E2f2* enhances *in vitro* axon regeneration. Our analysis highlights that transcription factor interaction and chromatin architecture play important roles as a regulator of axon regeneration.

## Introduction

Neurons within the central nervous system lack the intrinsic capacity to regenerate their axons after injury, leading to permanent functional deficits. In contrast, peripheral sensory neurons with cell soma in the dorsal root ganglia (DRG) can switch to a regenerative state after nerve injury to enable axon regeneration and functional recovery. Defining how injured sensory neurons transition to a pro-regenerative state may suggest future therapeutic approaches to improve neuronal recovery following axon injury.

Sensory neurons project both a peripheral axon branch into peripheral nerves and a central axon branch through the dorsal root into the spinal cord, providing a unique model system to study the mechanisms that control the axon regeneration program. Lesion of the peripheral axon is followed by robust and successful regeneration, whereas outgrowth of the centrally projecting axons is weak and does not lead to functional recovery. Multiple studies have utilized this differential response to gain insight into the early transcriptional events associated with successful regeneration (Smith and Skene, 1997; Stam et al., 2007; Blackmore, 2012; Mahar and Cavalli, 2018). A growing number of transcription factors (TFs) have been functionally linked to axon growth and regeneration (Venkatesh and Blackmore, 2017; Mahar and Cavalli, 2018). TFs work both independently of and in concert with epigenetic modifiers to increase the expression of pro-regenerative genes after injury (Weng et al., 2016; Fawcett and Verhaagen, 2018; Mahar and Cavalli, 2018) and represent ideal targets for therapeutic treatment of CNS injury (Fagoe et al., 2014; Venkatesh and Blackmore, 2017). One of the transcription factors that has emerged in many studies is ATF3, which was shown to drive the injured state and to be necessary for axonal regeneration and functional recovery (Chandran et al., 2016; Renthal et al., 2020). However, while ATF3 overexpression can promote peripheral axon regeneration (Seijffers et al., 2007), ATF3 fails to do so in several models of CNS injury (Seijffers et al., 2006; Fagoe et al., 2015; Venkatesh et al., 2016). This may be due to the fact that differentially expressed genes containing an ATF3 binding motif and the epigenomic signatures are largely distinct after spinal cord injury compared to nerve injury (Palmisano et al., 2019; Ewan et al., 2021). Another possibility is that a combination of transcription factors may provide more robust regeneration than a single TF alone (Chandran et al., 2016; Wang et al., 2018; Venkatesh et al., 2021). While genetic manipulation of TFs is a promising strategy, no single TF will likely be sufficient to fully restore neuron-intrinsic growth potential, and multiple, functionally interacting factors will be needed.

Selecting the optimal combination of TF has remained a difficult challenge (Venkatesh and Blackmore, 2017). One reason for this is that previous sequencing and bioinformatics studies have used whole DRG as input for sequencing (Michaelevski et al., 2010; Puttagunta et al., 2014; Chandran et al., 2016; Palmisano et al., 2019). The DRG is a highly heterogeneous cell population that consists of several cell types, including neurons, satellite glial cells, and macrophages (Kwon et al., 2013; Niemi et al., 2013; Avraham et al., 2020), limiting downstream bioinformatics analysis (Omura et al., 2015; Chandran et al., 2016; Tedeschi et al., 2016; Palmisano et al., 2019; Shin et al., 2019). Single-cell approaches have been used to unravel the transcriptional response of sensory neurons to nerve injury *in vivo* (Renthal et al., 2020), but the limited depth of sequencing also limits the analysis of the TFs implicated in the regeneration program.

To address this challenge, we characterized an *in vitro* model of embryonic DRG neurons that is widely used for studies of axon injury responses (Miller et al., 2009; Sasaki et al., 2009, 2020; Cho and Cavalli, 2012; Cho et al., 2013, 2015; Oh et al., 2018; Bloom et al., 2022). RNA-sequencing of eDRG cultures treated with a mitotic inhibitor revealed that a week after plating, these cultures are highly enriched in sensory neurons, and contain some neural progenitors but do not contain non-neuronal cells. Comparison to an *in vivo* single-cell data set of DRG cells traversing the primary sensory neuron lineage (Sharma et al., 2020) indicated that neurons in these eDRG cultures remained at an embryonic stage with all neuronal subtypes represented. Analysis of differentially expressed genes after injury revealed downregulation of pathways related to ion channels and upregulation of known regeneration-associated genes, including *Jun, Atf3*, and *Rest*, paralleling the axon injury response *in vivo* (Broude et al., 1997; Tedeschi et al., 2016; Lisi et al., 2017; Oh et al., 2018; Renthal et al., 2020). Analysis of TFs binding sites in differentially expressed genes revealed other known transcription factors promoting axon regeneration, such as *Myc* (Belin et al., 2015), *Hif1a* (Cho et al., 2015), *Pparg* (Lezana et al., 2016), *Ascl1a* (Williams et al., 2015), *Srf* (Stern et al., 2013), *Ctcf* (Palmisano et al., 2019), as well as other transcription factors not yet characterized in axon regeneration. We tested if overexpression of candidate pro-regenerative TFs alone or in combination promotes axon regeneration *in vitro*. Our results demonstrate that the expression of *Ctcf*, which is also known for its role in chromatin three-dimensional organization, does not enhance axon regeneration. However, a combination of *Ctcf* with *Yy1* or *E2f2* promotes *in vitro* axon regeneration. Our analysis reveals that pairs of TFs can functionally synergize to promote axon regeneration and highlights that TF interactions and chromatin architecture are essential regulators of axon regeneration.

## Results

### The eDRG spot culture model contains mostly neurons

**FIGURE 1:**
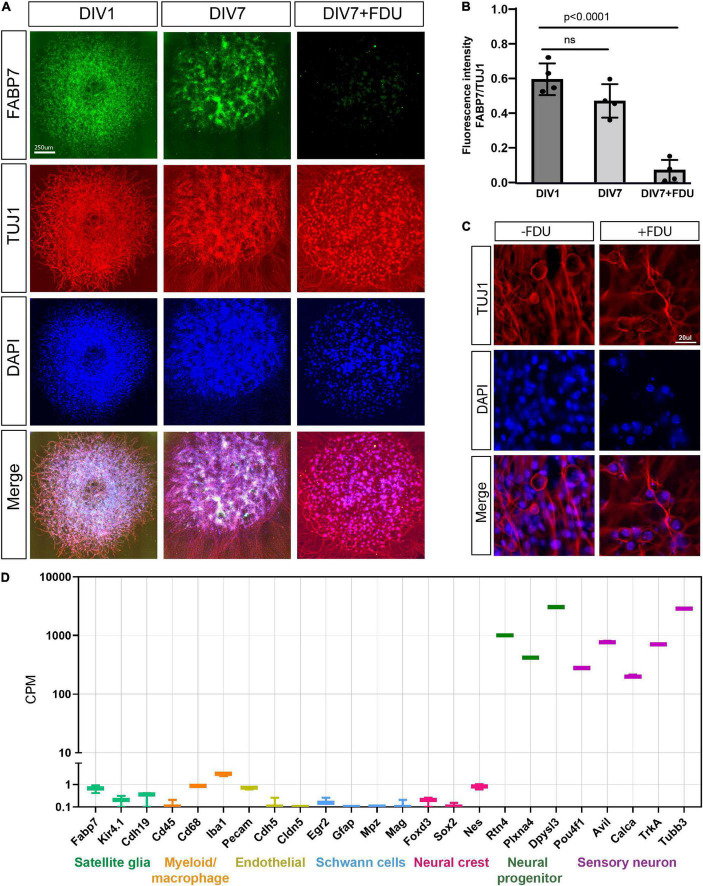
eDRG spot culture contains mostly sensory neurons after treatment with FDU. **(A)** Representative images of eDRG culture stained with the neuronal marker TUJ1 (red) and the Satellite glia marker FABP7 (green) at DIV1 and at DIV7 with and without treatment with FDU. Scale Bar: 250 μm. **(B)** Quantification of the fluorescence intensity of FABP7 normalized to TUJ1 at DIV1 and DIV7 ± FDU. *n* = 4 biologically independent animals. *P* values are determined by One way ANOVA. Data are presented as mean values ± SEM. **(C)** Representative images of eDRG culture in high magnification, with and without FDU, stained with TUJ1 (red) and DAPI (blue). Scale Bar: 20 μm. **(D)** Quantification of selected neuronal and non-neuronal markers (counts per million) from RNAseq analysis of eDRG spot culture at DIV7 treated with FDU.

The *in vitro* model of embryonic DRG neurons is widely used for studies of axon injury responses related to both axon degeneration and axon regeneration (Miller et al., 2009; Sasaki et al., 2009, 2020; Cho and Cavalli, 2012; Cho et al., 2013, 2015; Oh et al., 2018; Bloom et al., 2022). Yet, what type of neurons and what developmental stage is present in this model have not been examined in detail. In this culture model, embryonic day 13.5 DRG are spot cultured in the presence of the mitotic inhibitor 5-fluorodeoxyuridine (FDU). To determine the purity of these cultures, we stained for satellite glial cells, the most abundant cell type in DRG, with antibodies to FABP7 (Avraham et al., 2020). We observed that at days *in vitro* 1 (DIV1), FABP7 positive glial cells are present in high numbers (Figures 1A,B) but are nearly absent by DIV7 (Figures 1A,B), consistent with our previous results (Avraham et al., 2020). FDU treatment eliminates non-neuronal proliferating cells from the culture, without affecting neuronal properties and viability (Lesslich et al., 2022). Consistently, we did not observe pycnotic nuclei in our FDU-treated cultures compared to no FDU. We also did not detect any differences in neuronal morphology or axon elongation between the cultures that were treated with or without FDU (Figure 1C and Supplementary Figure 1). We next collected the mRNA from DRG spot cultures at DIV7 and submitted them for RNA sequencing. We examined the expression level of cell type-specific marker genes: satellite glial cells (*Fabp7, Kir4.1*, and *Cdh19*), myeloid/macrophages (*Cd45, Cd68*, and *Iba1*), endothelial (*Pecam, Chh5*, and *Cldn5*), Schwann cells (*Egr2, Gfap*, and *Mpz*), neural crest (*Foxd3, Sox2*, and *Nes*), neural progenitor markers (*Rtn4, Plxna4*, and *Dpysl3*) and sensory neurons (*Avil, Calca, TrkA*, and *Tubb3*). Compared to the average expression of sensory neuron marker genes and neural progenitor marker genes, the expression of non-neuronal cells marker genes and neural crest genes were all very low, indicating that these non-neuronal cell types are very low or absent in our preparation (Figure 1D). We conclude that the presence of FDU causes the progressive death of essentially all non-neuronal cells, resulting in a culture model that includes only sensory neurons and neural progenitors after 7 days *in vitro*.

### The eDRG spot culture model contains most neuronal subtypes present at E15.5 *in vivo*

**FIGURE 2:**
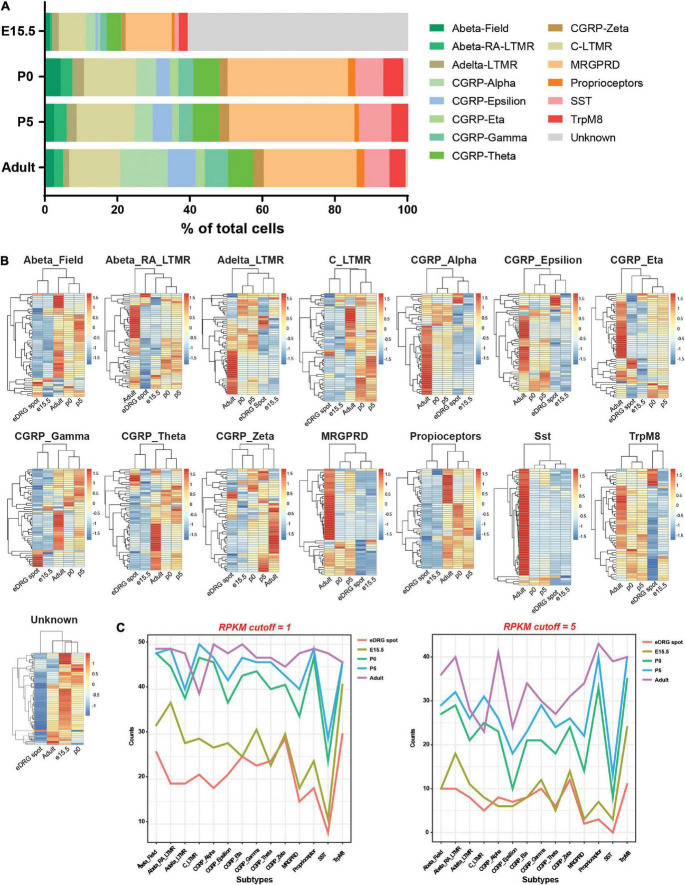
Neurons in the eDRG at DIV7 are similar to DRG neurons at the E15.5 developmental stage. **(A)** Fraction of 15 neuronal subtypes across four developmental stages of DRG neurons. **(B)** Heatmap shows the scaled *z*-score of the average CPM of 50 subtype-specific genes from eDRG controls and 4 developmental stages. Both row and column clustering were applied. High and low expressions are indicated in red and blue, respectively. **(C)** Plots of the counts of 50 subtype-specific genes expressed in each neuronal subtype during development using RPKM with cutoffs of 1 and 5.

Using this model, we next sought to understand whether neurons mature *in vitro* or remain at an unspecified stage and whether neuronal subtypes are present. Indeed, sensory neurons represent a heterogenous neuronal population that is specified during development in part by target-derived specific cues, such as NGF. In the eDRG spot culture model (eDRG spot), neurons are collected at E13.5 and cultured for 7 days in the presence of NGF. We thus compared the transcriptional profile of eDRG 7 days after plating to the transcriptomic atlas of cells traversing the somatosensory neuron lineage in mice (Sharma et al., 2020). We obtained the data from Sharma et al. for DRG at four developmental stages: E15.5, when innervation of peripheral and central target fields occurs; P0 when the maturation of sensory nerve ending with skin and other organs occurs and P5, when most peripheral endings mature into morphological states and central projection terminals are properly organized within select spinal cord laminae, and adult (P28-P42) (Sharma et al., 2020). From the single cell data, 15 neuronal subtypes were defined across these four developmental stages (Figure 2A and Supplementary Table 1) and each subtype was defined by the 4 most expressed genes (Supplementary Table 2). Fifty subtype-specific genes were then identified using the FindMarker function in Seurat (Supplementary Table 3). All genes were spot-checked by overlaying the expression levels on the t-SNE plot to ensure the computational method was correctly identifying genes with the prescribed features. We then combined read counts per subtypes, including scRNAseq and our eDRG spot RNAseq. The data were normalized with respect to library size and count differences were minimized between samples. We then calculated counts per million (CPM) and reads per kilobase million (RPKM) values and generated heat maps for the 50 enriched genes in each subtype. eDRG spot clustered closely to E15.5 in 11 out of 15 subtypes (Figure 2B). eDRG spot clustered with CGRP-Zeta from both E15.5 and P0 and with AbetaRA-LTMR and CGRP_Eta at E15.5, P0, and P5 (Figure 2B). Interestingly, the eDRG spot did not cluster with the unknown subtype (Figure 2B). This unknown subtype does not contain neural crest markers in the top 50 DEGs but expresses some neural progenitor genes, such as *Mllt11, Tuba1a, Stmn1, and Stmn2* (Supplementary Table 3). The neural progenitor genes expressed in the eDRG spot (Figure 1D) may reflect a different population of neural progenitors compared to the *in vivo* unknown cluster or represent a different developmental stage of this cluster. We also visualized the expression of genes in each neuronal subtype during development using RPKM with two different cut-offs (Figure 2C). These data further indicate that the eDRG spot model is most similar to E15.5 DRG *in vivo* and that most neuronal subtypes are present. These results suggest that while the eDRG spot is an embryonic culture, their stage at DIV7 is in between E17.5, in which axon growth *in vitro* is decreased and synapses have already formed *in vivo*, and E12.5, in which axon growth is active and synaptogenesis has not yet occurred (Prasad and Weiner, 2011; Tedeschi et al., 2016).

### Transcriptional changes are induced by *in vitro* axotomy over time

**FIGURE 3:**
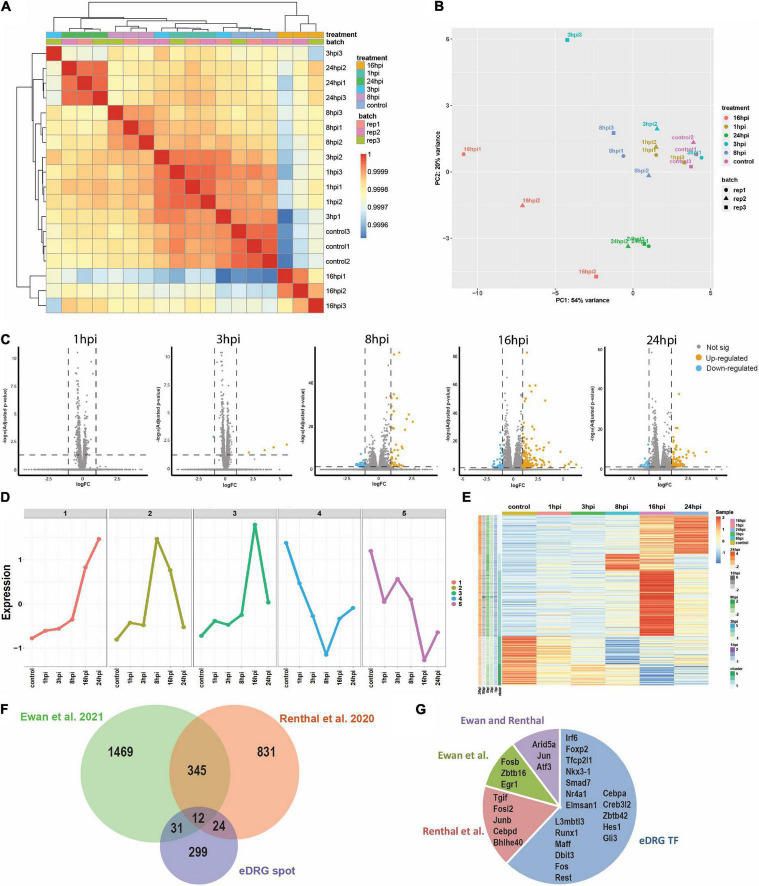
Time course analysis (1, 3, 8, 16, and 24 h) of the transcriptional response to injury in eDRG spot culture. **(A)** Heatmap of correlation of samples from time-course RNAseq analysis after axotomy. **(B)** PCA analysis of time-course RNAseq analysis after axotomy illustrates the relative similarity between sample groups at control, 1, 3, 8, 16, and 24 hpi (3 replicates per time point). **(C)** Volcano plots of differentially regulated genes after injury (*p* adj < 0.05, FC > 2). **(D)** K-means clustering identified five unique gene profile clusters with similar gene expression dynamics according to the time-course gene expression data. **(E)** Heatmap shows the scale z-score of the average CPM of 365 differentially expressed genes from time-course data. Red and blue cells indicate relative gene upregulation and downregulation, respectively. Green cells indicate cluster types identified in D. the cells next to cluster type are genes that are significantly expressed at different time points between treated and control groups. **(F)** Venn diagram comparing the differentially expressed genes after injury in the eDRG spot culture and 2 datasets of *in vivo* adult injury using enriched neuronal populations. **(G)** Out of the 29 up-regulated TFs in the eDRG spot, 11 were also identified in adult DRG neurons following sciatic nerve crush.

To unravel the neuron-intrinsic transcriptional changes in DRG neurons that arise after axon injury, we cut the axons of DRG neurons at DIV7 with a blade to induce an injury response and collected mRNA at 1, 3, 8, 16, and 24 h post injury (HPI). Hierarchical clustering and PCA analysis with batch correction revealed that 16 and 24 HPI displayed the most significant changes (Figures 3A,B). We identified a total of 467 differentially expressed genes (DEG), with most changes occurring 8–24 HPI (> 2-fold change, Padj < 0.05) and most DEG genes being upregulated (Figure 3C and Supplementary Table 4). Among the DEG, we identified 5 unique gene profile clusters with similar expression dynamics over time post-injury (Figures 3D,E and Supplementary Table 5). Gene ontology (GO) analysis of each cluster revealed unique biological pathways (Supplementary Figure 2). Clusters 4 and 5, in which genes are downregulated after injury, revealed enrichment for pathways related to ion channel activity, neurotransmitter, and synapse. These results parallel those of previous reports of the axon injury response *in vivo*, which demonstrated that ion channels are downregulated after injury, a process required for PNS regeneration (Tedeschi et al., 2016; Lisi et al., 2017; Oh et al., 2018). Pro-regenerative TFs known for their role *in vivo*, such as *Jun*, *Atf3*, and *Rest*, were found in clusters 2 and 3, in which expression peaked at 8 and 16 HPI, respectively. Cluster 2 and cluster 3 were enriched for pathways related to transcription factor, DNA binding, MAPK signaling, p53 signaling, and Wnt signaling, which are also known to regulate axon regeneration *in vivo* (Smith and Skene, 1997; Di Giovanni et al., 2006; Shin et al., 2012; Mahar and Cavalli, 2018). To further determine if the DEG elicited by *in vitro* axotomy is related to the *in vivo* situation, we compared our results to two recent studies that identified DEG in adult mice sensory neurons following sciatic nerve crush injury (Renthal et al., 2020; Ewan et al., 2021). We found 67 genes that were shared between the eDRG spot and at least one of the *in vivo* injury datasets (Figure 3F and Supplementary Table 4). To further determine if known pro-regenerative TFs are expressed in the eDRG spot model, we examined the up-regulated DEG and found that 29 were TFs, with 10 of them showing up-regulation at 8, 16, and 24 HPI (Table 1). These included TF known for their pro-regenerative function, such as *Rest*, *Jun*, and *Atf3*, paralleling the axon injury response *in vivo* (Broude et al., 1997; Tsujino et al., 2000; Mahar and Cavalli, 2018; Oh et al., 2018; Carlin et al., 2019; Renthal et al., 2020). We found that 11 of the 29 TFs were also differentially expressed in adult sensory neurons following nerve injury (Figure 3G and Table 1). To identify TFs that may regulate the expression of the upregulated DEG at 8, 16, and 24 HPI, we used an in-house algorithm that combines different analysis methods to predict TF binding sites (MORA, oPOSSUM3, and HOMER) (Zhao et al., 2007). We found a total of 43 TFs that were predicted in at least 2 different analysis methods (Table 2). These included previously characterized TFs, such as *Myc (Belin et al., 2015), Hif1a* (Cho et al., 2015), *Pparg* (Lezana et al., 2016), *Ascl1a* (Williams et al., 2015), *Srf* (Stern et al., 2013) and *Ctcf* (Palmisano et al., 2019), as well as other transcription factors not yet characterized in axon regeneration.

**TABLE 1:** TFs that were differentially upregulated at different hours after *in vitro* axon injury.

TFs	Domain	Condition
Arid5a	ARID	8/16 hpi
Tfcp2l1	CP2	16 hpi
Foxp2	Fork_head	16 hpi
Nkx3-1	Homeobox	16 hpi
Tgif1	Homeobox	8/16/24 hpi
Irf6	IRF	16 hpi
Smad7	MH1	16 hpi
Nr4a1	NGFIB-like	16/24 hpi
Elmsan1	Others	8 hpi
L3mbtl3	Others	16 hpi
Runx1	Runt	16 hpi
Jun	TF_bZIP	8 hpi
Maff	TF_bZIP	8 hpi
Fosl2	TF_bZIP	8/16 hpi
Ddit3	TF_bZIP	8/16 hpi
Fosb	TF_bZIP	8/16/24 hpi
Fos	TF_bZIP	8/16/24 hpi
Atf3	TF_bZIP	8/16/24 hpi
Junb	TF_bZIP	16 hpi
Cebpa	TF_bZIP	16 hpi
Creb3l2	TF_bZIP	16 hpi
Cebpd	TF_bZIP	16 hpi
Zbtb16	ZBTB	16 hpi
Zbtb42	ZBTB	16/24 hpi
Hes1	bHLH	8 hpi
Bhlhe40	bHLH	8 hpi
Egr1	zf-C2H2	8/16 hpi
Gli3	zf-C2H2	16 hpi
Rest	zf-C2H2	16 hpi

**TABLE 2:** TFs prediction binding sites of differentially up-regulated genes at 8, 16, and 24 HPI.

8 hpi	TF_Name	MORIA	oPOSSUM	homer	Family_Name	Num_Yes	Rank
1	STAT1	1	1	1	STAT	3	1
2	ARNT	1	1	0	BHLH	2	2
3	EGR1	1	1	0	C2H2 ZF	2	2
4	EGR2	1	0	1	C2H2 ZF	2	2
5	ELK1	1	1	0	ETS	2	2
6	FEV	1	1	0	ETS	2	2
7	GABPA	1	1	0	ETS	2	2
8	GATA1	0	1	1	GATA	2	2
9	HIF1A	1	1	0	BHLH	2	2
10	INSM1	1	1	0	C2H2 ZF	2	2
11	IRF2	1	0	1	IRF	2	2
12	JUN	1	1	0	BZIP	2	2
13	MYC	1	1	0	BHLH	2	2
14	MZF1	1	1	0	C2H2 ZF	2	2
15	NFE2L2	1	1	0	BZIP	2	2
16	NR4A2	1	1	0	NUCLEAR RECEPTOR	2	2
17	PAX5	1	0	1	PAIRED BOX	2	2
18	PLAG1	1	1	0	C2H2 ZF	2	2
19	RELA	1	1	0	REL	2	2
20	RORA	1	1	0	NUCLEAR RECEPTOR	2	2
21	RXRA	1	1	0	NUCLEAR RECEPTOR	2	2
22	SP1	1	1	0	C2H2 ZF	2	2
23	SRF	1	1	0	MADS BOX	2	2
24	YY1	0	1	1	C2H2 ZF	2	2

**16 hpi**	**TF_Name**	**MORIA**	**oPOSSUM**	**homer**	**Family_Name**	**Num_Yes**	**Rank**

1	AR	1	1	0	NUCLEAR RECEPTOR	2	1
2	ARNT	1	1	0	BHLH	2	1
3	CTCF	1	1	0	C2H2 ZF	2	1
4	E2F2	1	1	0	E2F	2	1
5	EBF1	1	1	0	BHLH	2	1
6	EGR1	1	1	0	C2H2 ZF	2	1
7	ESR1	1	1	0	NUCLEAR RECEPTOR	2	1
8	HIF1A	1	1	0	BHLH	2	1
9	HINFP	1	1	0	C2H2 ZF	2	1
10	HNF4A	1	1	0	NUCLEAR RECEPTOR	2	1
11	INSM1	1	1	0	C2H2 ZF	2	1
12	IRF2	1	1	0	IRF	2	1
13	JUN	0	1	1	BZIP	2	1
14	NFKB1	1	1	0	REL	2	1
15	NR3C1	1	1	0	NUCLEAR RECEPTOR	2	1
16	PAX5	1	1	0	PAIRED BOX	2	1
17	PLAG1	1	1	0	C2H2 ZF	2	1
18	PPARG	1	1	0	NUCLEAR RECEPTOR	2	1
19	RELA	1	1	0	REL	2	1
20	RXRA	1	1	0	NUCLEAR RECEPTOR	2	1
21	SP1	1	1	0	C2H2 ZF	2	1
22	SPI1	1	1	0	ETS	2	1
23	SPIB	0	1	1	ETS	2	1
24	SRF	1	1	0	MADS BOX	2	1
**24 hpi**	**TF_Name**	**MORIA**	**oPOSSUM**	**homer**	**Family_Name**	**Num_Yes Rank**

1	ARNT	1	1	1	BHLH	3	1
2	ATF7	1	0	1	BZIP	2	2
3	CREB1	1	1	0	BZIP	2	2
4	E2F1	1	1	0	E2F	2	2
5	EBF1	1	1	0	BHLH	2	2
6	EGR1	1	1	0	C2H2 ZF	2	2
7	ESRRB	1	1	0	NUCLEAR RECEPTOR	2	2
8	INSM1	1	1	0	C2H2 ZF	2	2
9	JUN	1	1	0	BZIP	2	2
10	MYOG	1	0	1	BHLH	2	2
11	PAX5	1	1	0	PAIRED BOX	2	2
12	PLAG1	1	1	0	C2H2 ZF	2	2
13	RXRA	1	1	0	NUCLEAR RECEPTOR	2	2
14	SPI1	1	1	0	ETS	2	2
15	VDR	1	1	0	NUCLEAR RECEPTOR	2	2
16	YY1	1	1	0	C2H2 ZF	2	2
17	ZFP423	1	1	0	C2H2 ZF	2	2

### Lentiviral-based expression of pro-regenerative TF identifies TF combination that improves axon regeneration *in vitro*

Because it is likely that a single TF is not sufficient to fully restore neuron-intrinsic axon growth potential, and that a combination of TFs is needed (Venkatesh and Blackmore, 2017), we decided to overexpress combinations of TFs identified in our analysis, and test if a given TF combination can promote regeneration. We used the eDRG spot culture model to screen the effect of lentiviral mediated expression of TFs combinations on regenerative axon growth 24 h after *in vitro* axotomy, as described previously (Cho et al., 2013, 2015; Avraham et al., 2020). We selected 8 TFs that were predicted to regulate DEG after injury in our analysis and were also identified in different *in vivo* regeneration models but have not been directly tested for their pro-regenerative potential. These are *Atf3* (Tsujino et al., 2000; Chandran et al., 2016; Renthal et al., 2020), *Fos* (Stam et al., 2007; Michaelevski et al., 2010), *Egr1* (Zou et al., 2009; Michaelevski et al., 2010), *Nfya* (Smith et al., 2011), *Ebf1* (Smith et al., 2011), *E2f2* (Pita-Thomas et al., 2021), *Ctcf* (Palmisano et al., 2019; Pita-Thomas et al., 2021), *Yy1* (Stam et al., 2007). Interestingly, CTCF is also known as a chromatin remodeler and conditional deletion of CTCF *in vivo* impairs nerve regeneration, implicating chromatin organization in the regenerative competence (Palmisano et al., 2019). Whether CTCF expression is sufficient to enhance axon regeneration had not been tested.

**FIGURE 4:**
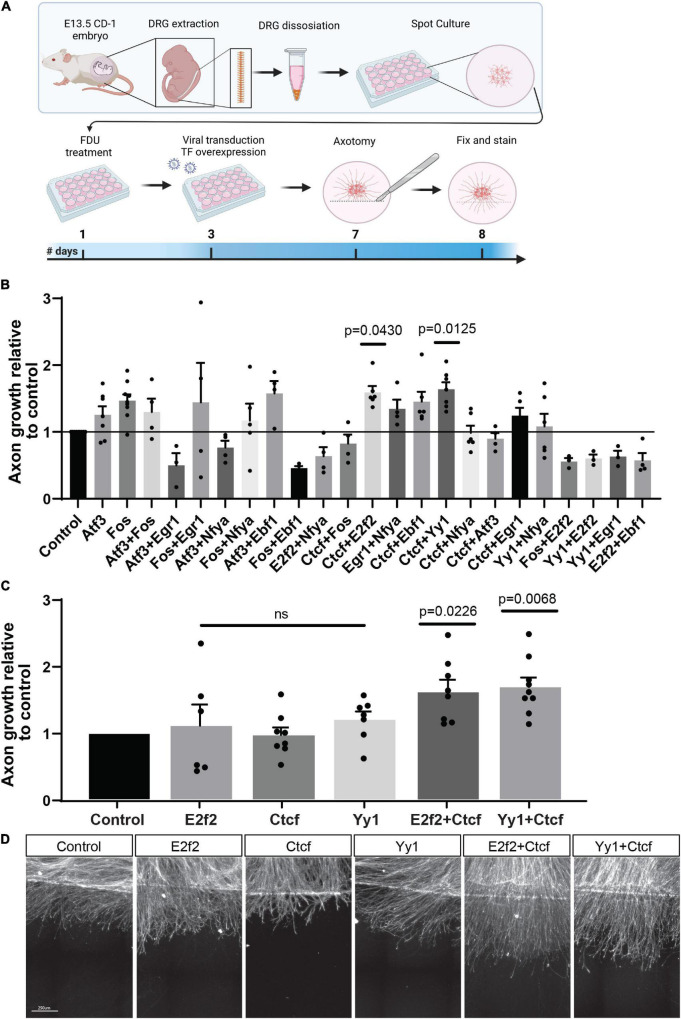
Overexpression of TF combinations in eDRG axotomy model. **(A)** Schematic timeline of the experimental procedure for TF overexpression in injured cultured eDRG neurons. **(B)** Axon growth of infected cells with TF combinations relative to control. **(C)** Significant TF were tested alone and repeated in combinations. **(D)** Represented images of axotomized cultures infected with the pro-regenerative TFs alone and in combinations. Scale Bar: 250 μm. *n* = 3–9 biologically independent animals examined over 2 independent experiments *P*-values determined by one-way ANOVA followed by Dunnett’s multiple comparisons test. Data are presented as mean values ± SEM.

eDRG spot cultured neurons were infected with lentiviruses expressing the selected TFs in pairwise combination 4 days before axotomy (Figure 4A). Axons were injured at DIV7, immunostained for the regeneration marker SCG10 24 h later and, analysis for axon growth past the injury site was performed as described previously (Figure 4A; Cho et al., 2015; Avraham et al., 2020). Axonal length after treatment with an expression of TF combinations was normalized to lentivirus expressing mCherry as a control. We verified that in these conditions, transduction efficiency was high by detection of mCherry in > 90% of neurons (Supplementary Figure 3). We observed that a combination of the TFs *Ctcf* with *Yy1* or *E2f2* had a significant effect on regenerative axon growth (Figures 4B,C), but there was no significant increase in axon growth when *Ctcf*, *E2f2*, or *Yy1* were expressed alone (Figures 4C,D). These results identify *Ctcf*, *Yy1*, and *E2f2* as new pro-regenerative TFs and highlight that TF interaction and chromatin remodeling play an important role as a regulator of axon regeneration.

## Discussion

In the present study, we used RNA-sequencing and bioinformatic analyses to identify pairs of TFs that synergize to promote axon regeneration. Our analysis provides a framework for the development of combinatorial gene over-expression approaches which may provide strategies to induce axon regeneration *in vivo.* We also characterized the eDRG spot culture model and determined that this model partially mimics the *in vivo* injury responses.

Our data suggest that the eDRG spot culture is a useful model to identify genes and transcription factors that regulate the neuron’s intrinsic capacity for axon regeneration. Our analysis reveals that this model contains sensory neurons that are in a stage between E12.5, in which axon growth is active and synaptogenesis has not occurred *in vivo*, and E17.5, in which axon growth *in vitro* is decreased and synapses have already formed *in vivo*. The DEG elicited by *in vitro* injury parallels in part those observed *in vivo*. However, this model does present limitations, given that non-neuronal cells are not present and thus contributions from satellite glial cells (Avraham et al., 2020, 2021), immune cells (Niemi et al., 2013) or other extrinsic factors, such as the microbiome (Serger et al., 2022), are not contributing to the neuronal response to injury. This model also does not allow to study the age-dependent neuronal regenerative decline (Zhou et al., 2022). The strengths of this model lie in the ability to study intrinsic neuronal mechanisms that regulate axon growth capacity in a defined and characterized neuronal population that are accessible to genetic and pharmacological manipulations.

Previous studies have taken different approaches to identify regulators of axon regeneration, including high throughput phenotypic screening (Simpson et al., 2015; Huebner et al., 2018; Karney-Grobe et al., 2018; Sekine et al., 2018) and transcriptional-based approaches (Michaelevski et al., 2010; Puttagunta et al., 2014; Cho et al., 2015; Omura et al., 2015; Chandran et al., 2016). Each method has provided key insight into the molecular mechanisms of regeneration and identified targets that can promote some axon regeneration. ATF3 is a key TF inducing the pro-regenerative state in sensory neurons and loss of ATF3 impairs axon regeneration (Tsujino et al., 2000; Seijffers et al., 2007; Renthal et al., 2020). Overexpression of ATF3 alone or in combination with c-jun was shown to improve DRG axon growth *in vitro* (Chandran et al., 2016) and had only a mild effect on cortical neuron neurite outgrowth *in vitro* (Simpson et al., 2015). However, ATF3 expression fails to promote robust axon regrowth in several models of CNS injury (Seijffers et al., 2006; Fagoe et al., 2015; Venkatesh et al., 2016). This may be due to the fact that differentially expressed genes containing an ATF3 binding motif and the epigenomic signatures are largely distinct after spinal cord injury compared to nerve injury (Palmisano et al., 2019; Ewan et al., 2021). Indeed, several epigenetic mechanisms implicating histone deacetylase (HDAC3 and HDAC5) and histone acyltransferase (PCAF) operate after peripheral but not central axon injury (Cho and Cavalli, 2012; Finelli et al., 2013; Puttagunta et al., 2014; Hervera et al., 2019). The combination of TFs expression with epigenetic modifiers might synergize to stimulate long-range axon regeneration.

Recent epigenomics studies revealed that neuronal conditional deletion of CTCF impaired nerve regeneration, implicating chromatin organization in the axon regenerative competence (Palmisano et al., 2019). Whether CTCF expression could promote axon regeneration had not been tested. CTCF is known as a chromatin remodeler with a critical role in connecting higher-order chromatin folding in pluripotent stem cells (Beagan et al., 2017). Our results suggest that CTCF expression in combination with E2F2 or YY1, but not alone, stimulates axon growth *in vitro*. This co-factor requirement appears specific since CTCF did not synergize with NFYA, FOS, ATF3, EGR1, or EBF1 in our assay or with KLF6 in a cortical growth assay (Venkatesh et al., 2021). In the context of genome imprinting, YY1 is a required cofactor for CTCF function in the X chromosome binary switch (Donohoe et al., 2007). Many clustered YY1 and CTCF binding sites are conserved among humans, mice, and cows (Kang et al., 2009). YY1 contributes to enhancer-promoter structural interactions in a manner analogous to DNA interactions mediated by CTCF (Weintraub et al., 2017), suggesting that enhancer-promoter looping facilitates gene expression required for axon regeneration. This may also underlie why combined TF expression without chromatin remodeler can increase collateral sprouting but not long-range regenerative growth through sites of the spinal lesion (Venkatesh et al., 2021). Indeed, prior work shows that the epigenetic landscape of pro-growth genes acquires marks of heterochromatin and transcriptional repression with age (Venkatesh et al., 2016). Reactivating the pro-growth program will require changes in the epigenomic landscape. Our algorithm that combines different analysis methods to predict TF binding sites provides useful insights into the selection of TF combinations and suggests that CTCF represents a potent target for those combinations. In combination with previous bioinformatics analyses of axon regeneration (Chandran et al., 2016; Venkatesh and Blackmore, 2017), our results will help guide future experiments to select optimal TFs combinations for promoting axon regeneration.

## Materials and methods

### Animals and procedures

All animal procedures were performed in accordance with the WashU animal care committee’s regulations. Time pregnant e13.5 CD-1 mice were used for all experiments.

### Embryonic DRG cultures and regeneration assay

Dorsal root ganglia were isolated from time pregnant e13.5 CD-1 mice into dissection media consisting of DMEM and Pen/Strep. After a short centrifugation, dissection media was aspirated and cells were digested in 0.05% Trypsin-EDTA for 25 min. Next, cells were pelleted by centrifuging for 2 min at 500 × *g*, the supernatant was aspirated, and Neurobasal was added. Cells were then triturated 25x and added to the growth medium containing Neurobasal, B27 Plus, 5 μM FDU, 1 ng/ml NGF, Glutamax, and Pen/Strep. Approximately 10,000 cells were added to each well in a 2.5 μl spot. Spotted cells were allowed to adhere for 10 min before the addition of the growth medium. Plates were pre-coated with 100 μg/ml poly-D-lysine overnight and washed with sterile water prior to plating. For the regeneration assay, lentivirus was added on DIV3. Cells were then injured using an 8 mm microtome blade on DIV 7 and fixed 24 h later with 4% PFA. For immunostaining, wells were incubated in PBS-0.1% Triton (PBST) for 1 h at room temperature containing SCG10 primary antibody (Novus Bio NBP1-49461; RRID:AB_10011569). The wells were then washed 3x with PBST and then incubated in PBST solution containing fluorescent-labeled goat anti-rabbit secondary antibody (AlexaFluor-555; Invitrogen A21428; RRID:AB_141784) for 1 h at room temperature. Finally, wells were washed 3x with PBS. For immunostaining of spot culture at DIV1 and DIV7, wells were stained with TUJ1 primary antibody (Biolegend 802001; RRID:AB_2564645), Fabp7 (Thermo Fisher Scientific Cat# PA5-24949, RRID:AB_2542449) and DAPI (1:1,000).

### Viral production

To produce lentivirus, HEK293T cells were grown on 15 cm plates to a confluency of 70–90%. On DIV0, 3 μg PMD2.G, 9 μg PsPax2, and 12 μg of target plasmid were incubated for 15 min with 96 μg of PEI Max (Polysciences 24765-1) per 15 cm dish in Optimem at room temperature. Following incubation, the transfection solution was added dropwise to the plate, gently mixed, and incubated for 96 h. Following incubation, viral-containing supernatant was collected and centrifuged at 500 × *g* for 10 min to remove cellular debris. Cleared supernatants were further filtered with a 0.45 μm PES filter to remove the remaining debris. A total of 30 μl of viral-containing supernatant was added into 500 μl of media in each well in a 24-well plate. Virus with mCherry was used as a control for infection efficiency and for axon regeneration. Overexpression clones in the lentiviral backbone vector Plx304 were obtained from the human lentiviral ORF library (transOMICs).

**Table:** 

Gene symbol	Clone ID	Gene ID
ATF3	TOLH-1504621	467
FOS	TOLH-1506177	2353
CTCF	TOLH-1511000	10664
YY1	TOLH-1504348	7528
EGR1	TOLH-1518371	1958
NFYA	TOLH-1509753	4800
EBF1	TOLH-1517318	1879
E2F2	TOLH-1508827	1870
		

### RNA sequencing and bioinformatics analysis

For RNA sequencing experiments, embryonic dorsal root ganglia were injured with a blade, isolated at 1, 3, 8, 16, and 24 h post-injury. Cells were lysed and total RNA was extracted using the PureLink RNA Mini Kit (Thermo Fisher Scientific 12183018A) with on-column DNase treatment (Thermo Fisher Scientific 12185010). Next, RNA concentration was determined using a NanoDrop 2000 (Thermo Fisher Scientific). First-strand synthesis was then performed using the High Capacity cDNA Reverse Transcription kit (Applied Biosystems).

Samples were submitted to the Genome Access Technology Center at Washington University in St. Louis for library preparation and sequencing. Libraries were sequenced on an Illumina HiSeq2500 using 2 × 101 bp runs.

Reads were processed using an in-house pipeline and open-source R packages. Briefly, raw reads were first trimmed using cutadapt to remove low-quality bases and reads. Trimmed reads were then aligned to the mouse genome 10 mm with GENCODE annotation vM20 using STAR (v2.5.4) with default parameters. Transcript quantification was performed using featureCounts from the subread package (v1.6.3). further quality control assessments were made using RSeQC and RSEM, and the batch correction was performed using edgeR, EDASeq, and RUVSeq. Gene type and transcription factor (TF) annotation were performed using mouse GENCODE vM20 and AnimalTFDB, respectively.

Principle component analysis and differential expression analysis for neurons collected at 1, 3, 8, 16, and 24 h post injury (HPI) and control groups were determined using DESeq2 in negative binomial mode using batch-corrected transcripts from featureCounts (> 2-fold expression change, > 1 count per million (CPM), Benjamini corrected *P* < 0.05). Pairwise comparisons were made between time points vs control to determine differentially expressed genes (DEGs) within each group. To determine transcription factor binding site enrichment, the significantly upregulated genes were used as input for oPOSSUM 3.0 for TF enrichment analysis with a default cutoff for statistical significance^1^. TF enrichment was also confirmed using the prediction of TF binding sites (MORIA and HOMER). The HOMER (Heinz et al., 2010) and motif over-representation analysis (MORA) (Zhao et al., 2007) algorithms were used to identify transcription factor binding sites enriched in the upstream regions compared with background sequences in the genome. An in-house script was used to generate a combined ranking of predicted TFs.

### Image analysis

All images were acquired at 10x using a Nikon TE2000 microscope and image analysis was completed using Nikon Elements. For embryonic dorsal root ganglia experiments, regenerative length was measured from the visible blade mark to the end of the regenerating axons. Each technical replicate was measured 4–6 times and three technical replicates were measured per biological replicate.

### Statistical analysis

All experimenters were blinded to treatment conditions while performing image analysis. All statistical analysis was completed using GraphPad Prism. Data are presented as ± SEM. All statistical values are reported in the text as appropriate.

## Data availability statement

The datasets presented in this study can be found in online repositories. The name of the repository and accession number can be found below: National Center for Biotechnology Information (NCBI) Gene Expression Omnibus (GEO), https://www.ncbi.nlm.nih.gov/geo/, GSE138480.

## Ethics statement

The animal study was reviewed and approved by the Washington University School of Medicine Institutional Animal Care and Use Committee (IACUC) under protocol A-3381-01. All experiments were performed in accordance with the relevant guidelines and regulations. All experimental protocols involving mice were approved by the Washington University School of Medicine (protocol #21-0104 and #20-0173). Mice were housed and cared for in the Washington University School of Medicine animal care facility. This facility is accredited by the Association for Assessment and Accreditation of Laboratory Animal Care (AALAC) and conforms to the PHS guidelines for Animal Care. Accreditation - 7/18/97, USDA Accreditation: Registration #43- R-008.

## Author contributions

OA, JL, GZ, and VC conceived and designed the experiments. OA, JL, and KL performed the experiments. TL and GZ performed the bioinformatic analyses. OA and JL performed the data analysis. VC secured the funding. OA, GZ, and VC wrote the manuscript. All authors approved the submitted version.

## Footnotes

1
https://cisreg.ca/software/

## Supplementary material

Supplementary Figure 1 Representative images of eDRG spot culture at DIV7 at low magnification, with and without FDU, stained with TUJ1. Scale bar: 1,000 μm.

Supplementary Figure 2 Gene Ontology and KEGG pathway analysis (mouse 2019) for the different gene profile clusters with similar gene expression dynamics.

Supplementary Figure 3 eDRG spot culture transduced with a lentivirus expressing mCherry 3 days after infection.

Supplementary Table 1 Quantification of neuronal subtypes in the DRG at different developmental stages.

Supplementary Table 2 Four most expressed genes in each neuronal subtype.

Supplementary Table 3 50 neuronal subtype-specific genes (FindMarker, Seurat).

Supplementary Table 4 Differentially regulated genes at different time points after axotomy. (*p* adj < 0.05, FC > 2).

Supplementary Table 5 5 unique gene profile clusters with similar expression dynamics over time post-injury.
