# Supplementary figures and images for: Analysis of neuronal injury transcriptional response identifies CTCF and YY1 as co-operating factors regulating axon regeneration
Source: Front Mol Neurosci. 2022 Aug 23;15:967472. doi: 10.3389/fnmol.2022.967472 (PMC9446241; doi:10.3389/fnmol.2022.967472)

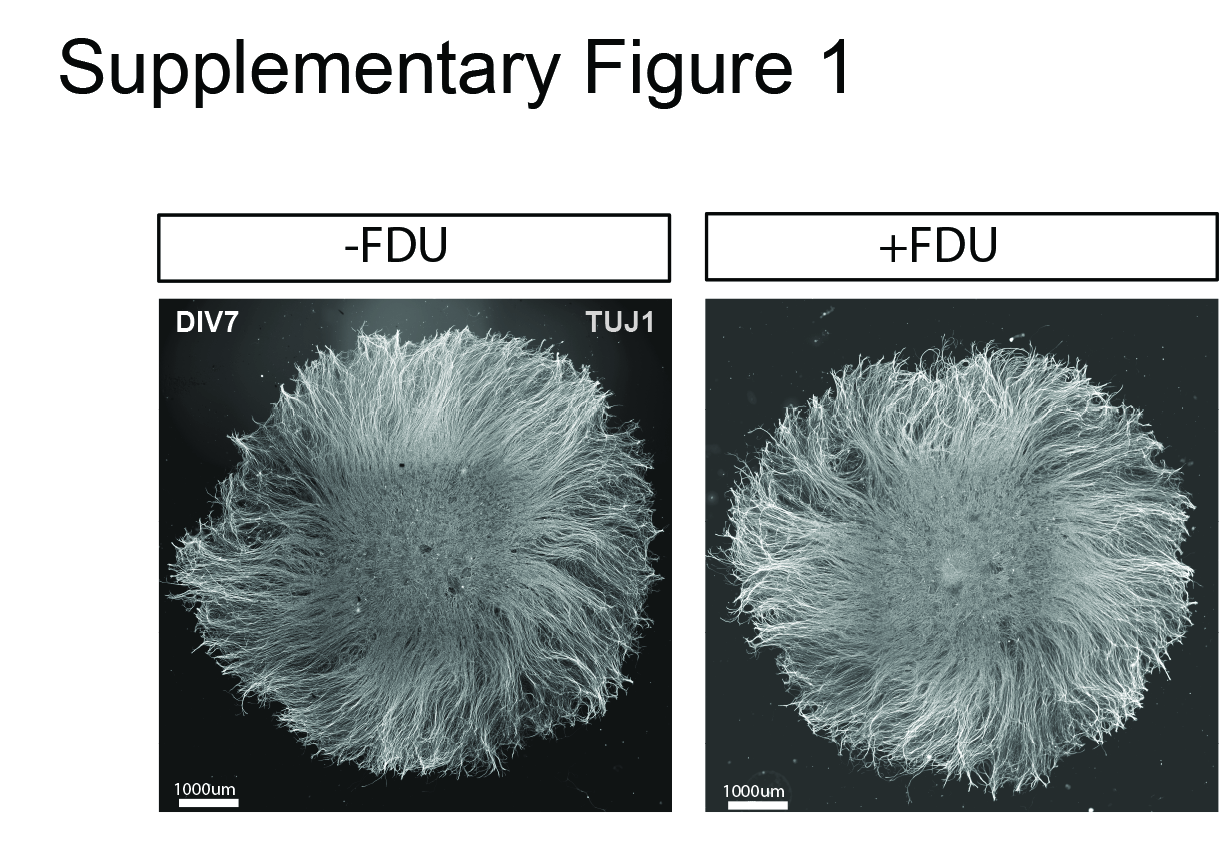

Supplement: Supplementary Figure 1 — Representative images of eDRG spot culture at DIV7 at low magnification, with and without FDU, stained with TUJ1. Scale bar: 1,000 μm. [file Image_1.TIF]

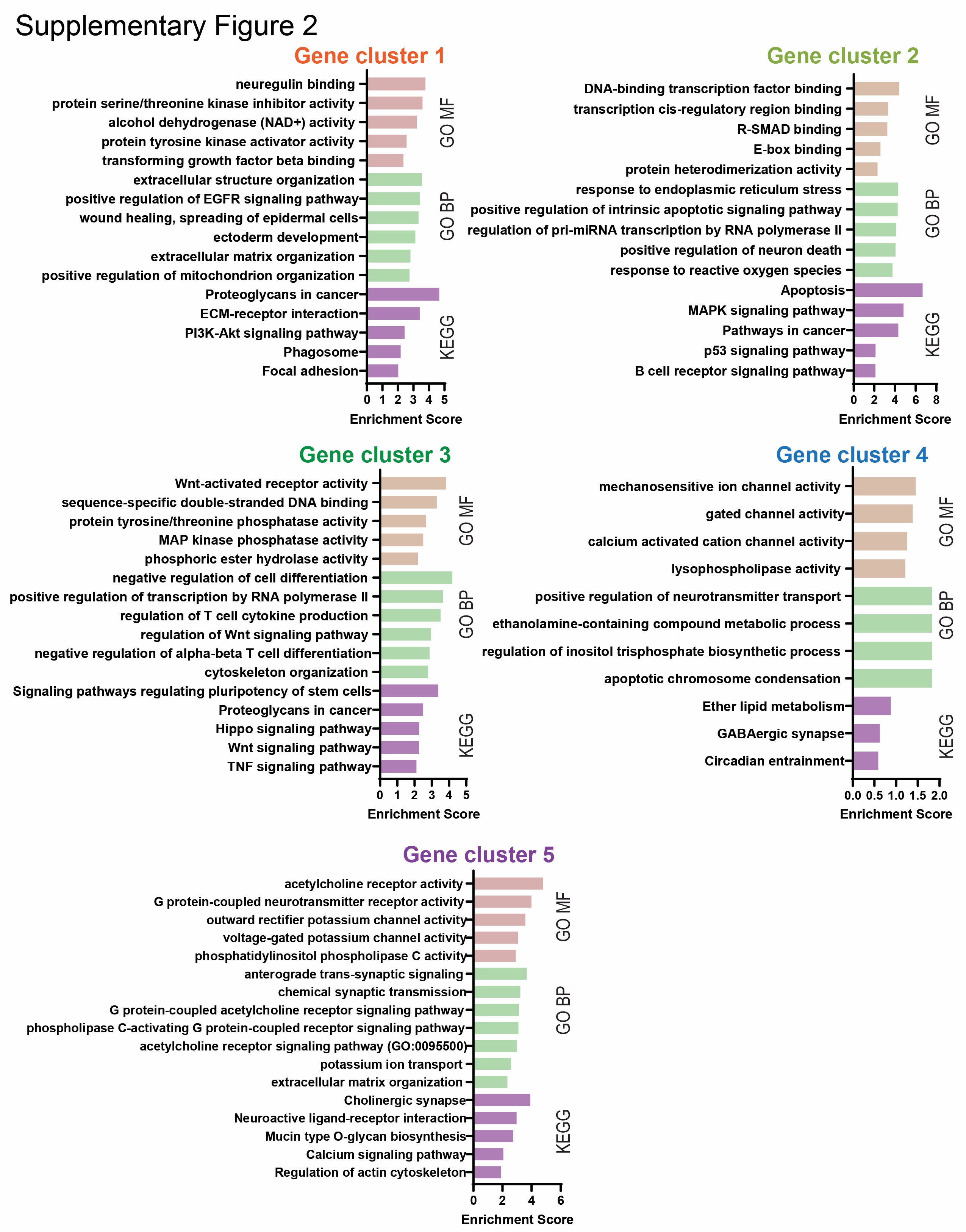

Supplement: Supplementary Figure 2 — Gene Ontology and KEGG pathway analysis (mouse 2019) for the different gene profile clusters with similar gene expression dynamics. [file Image_2.JPEG]

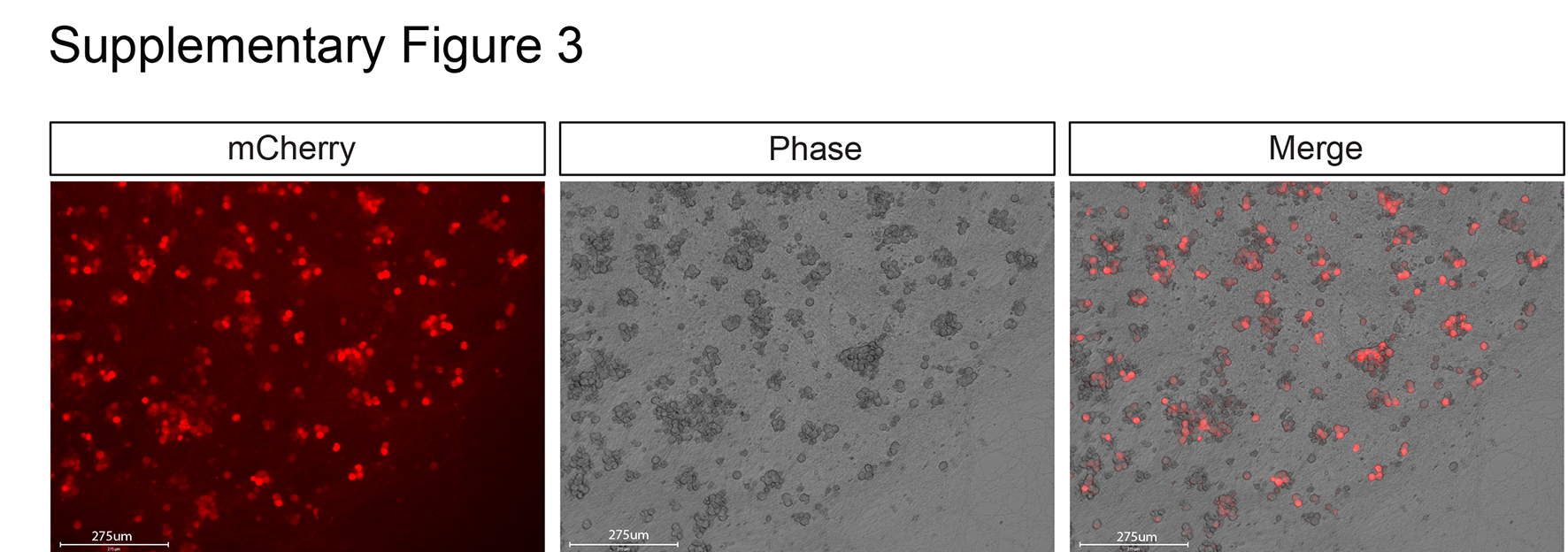

Supplement: Supplementary Figure 3 — eDRG spot culture transduced with a lentivirus expressing mCherry 3 days after infection. [file Image_3.TIF]
